# Effect of socioeconomic characteristics and lifestyle on BMI distribution in the Chinese population: a population-based cross-sectional study

**DOI:** 10.1186/s12889-021-11405-4

**Published:** 2021-07-10

**Authors:** Weihua Wang, Lin Qiu, Rina Sa, Shaonong Dang, Feng Liu, Xiang Xiao

**Affiliations:** 1 Department of Chronic Disease Control and Prevention, Shaanxi Provincial Center for Disease Control and Prevention, No. 3, Jiandong Road, Xi’an, People’s Republic of China; 2 Department of Epidemiology and Biostatistics, School of Public Health, Xi’an Jiaotong University Health Science Center, Xi’an, People’s Republic of China; 3 School of Public Health, University of Alberta, Edmonton, Canada

**Keywords:** Body mass index, Socioeconomic characteristics, Quantile regression, Lifestyle factors

## Abstract

**Background:**

Body mass index (BMI) is an accepted measurement that is widely used to quantify overweight and obesity at the population level. Previous studies have described the distribution variation of BMI through applying common statistical approaches, such as multiple linear or logistic regression analyses. This study proposed that associations between BMI and socioeconomic characteristics, diet, and lifestyle factors varied across the conditional BMI distribution.

**Methods:**

This study was based on a sample of 10,023 Chinese adults who participated in the monitoring of chronic diseases and associated risk factors in Shaanxi Province, Northwest China, in 2013. Cross-quantile factors were observed in the relationships between major risk factors and BMI through quantile regression (QR) and ordinary least squares (OLS) regression.

**Results:**

Participants’ mean BMI was 24.19 ± 3.51 kg/m^2^ (range 14.33–52.82 kg/m^2^). The QR results showed that living in urban areas was associated with BMI in the low and central quantiles (10th–60th). Participants with 6–9 years of education were 0.23–0.38 BMI units higher in the first half of the BMI quantiles compared with those with ≤6 years of education. There was a positive association between consumption of red meat and BMI; however, the association diminished from the 10th to the 50th quantile. Intake of oil and alcohol were positively associated with all BMI quantiles. Cigarette smoking per day was negatively associated with BMI, which showed a *U*-shaped distribution. The above results were also observed in the OLS.

**Conclusion:**

This study implies that in addition to socioeconomic characteristics, limiting oil and alcohol intake may decrease BMI score. Consuming more red meat could be a strategy to increase BMI.

## Background

The average body mass index (BMI) has increased worldwide in the past two decades, especially in low- and middle-income countries [1–4]. It is known that obesity defined by BMI is a risk factor for multiple non-communicable chronic diseases, such as cancer and cardiovascular disease, which places an increasing burden on healthcare systems [5, 6]. The global number of deaths caused by non-communicable chronic diseases is expected to rise from 59 to 69% between 2002 and 2030 [7, 8].

With the rapid social and economic development in China, BMI distribution curves moved to the right from 1981 to 2011, with an increasing prevalence of overweight and obesity in the Chinese population [3, 9]. The prevalence of obesity among Chinese adults more than doubled in the last three decades [10], and about 46% of Chinese adults are obese or overweight [11]. Although many studies have focused on obesity in China, there are noticeable demographic and socioeconomic disparities given the wide variation in China [12]. Behaviors such as tobacco smoking, alcohol intake, and physical activity have also been shown to be associated with individual weight gain or loss [13–15]. Most previous studies that examined the effect of health behaviors on changes in BMI used either multiple linear or logistic regression analyses, which do not capture distribution variations in different BMI quantiles [16–18]. Therefore, the former methods may mask some important relations in various quantiles of BMI distribution. In contrast, it has been suggested that quantile regression (QR) is a useful method to address important differences across the overall BMI distribution [19], and is more robust against skewness or outliers than traditional linear regression. Given that the effect of risk factors may vary by BMI distribution, we aimed to examine the effects of socioeconomic status, lifestyle factors, and other factors on different BMI status by QR. This enabled us to observe the effect of risk factors on BMI ranging from low to high quantiles along the entire BMI distribution.

## Methods

### Study setting

Shaanxi Province is located in Northwest China. It consists of three regions: Northern, Central, and Southern Shaanxi. Shaanxi is a less developed province in terms of socioeconomic factors compared with Eastern China. The provincial gross domestic product in 2019 was 365 billion US dollars, which was estimated in the middle of the 31 provincial regions in China [20]. The resident population of Shaanxi Province was 37.9 million people [21].

### Study design and sampling

Shaanxi Center for Disease Control and Prevention has implemented the monitoring of chronic diseases and associated risk factors every 3 years since 2004, as detailed in the China Chronic Disease Prevention and Control Work Plan. Because of the largest sample size of all surveys from 2004 to 2018, this study was based on data collected in 2013, which covered 17 monitoring points (counties or districts) with 10,300 participants recruited in Shaanxi Province. The target subjects were Chinese people aged 18 years and above who had lived for more than 6 months in their current residence. Participant recruitment was conducted by stratified multistage sampling. In the first stage of sampling, 10 counties were selected from Shaanxi Province using the method of probability proportional to size. In the second stage of sampling, four townships were selected in each selected county using the same method. In the third stage of sampling, three villages were selected from each selected township using the same method. In the fourth stage of sampling, at least 50 households were randomly selected from the selected villages. In the final stage of sampling, one adult aged 18 years and above was randomly selected from each household. A total sample of 10,320 participants was required, assuming an overweight prevalence of 30.6% in the study population, a relative error of 15%, a = 0.05, design effect = 2.0, $\mathrm{N}=\mathrm{deff}{\upmu}_{\upalpha}^2\frac{\mathrm{P}\left(1-\mathrm{P}\right)}{{\mathrm{d}}^2}$, and accounting for an expected 10% non-response rate [22].

## Ethics statement

The National Health and Family Planning Commission (NHFPC, previously the Ministry of Health) of China and the Ethics Committee of the Chinese Center for Disease Control and Prevention approved the program. An informed consent form was completed by all participants.

### Quality control

Interviewers in this study were staff from local CDC and healthcare institutes. Passing a test after training was mandatory before staff conducted the survey. The test aimed to examine each interviewer’s familiarity with the questionnaires, operation standards, and measurement of height, weight, blood pressure, and precautions for blood draw. Anthropometric measurements were standardized before the survey. A checking system that comprised checking in the field by interviewers themselves, officials from the local CDC, and supervisors from the workgroup was applied to control the quality of this study. Personnel from local healthcare institutes and health offices helped organize the field investigation and explained the study procedures to participants. Participants with cognitive problems, language problems, mental disorders, or severe diseases were excluded. Recruited participants were interviewed twice if logical inconsistencies and missing values were detected.

### Measures

Questionnaires were used to obtain information about participants’ demographic characteristics, dietary habits, lifestyle behaviors, disease history, and health status through face-to-face interviews conducted by trained local CDC staff. The questionnaire that was used in the survey was drafted by the China CDC and its reliability and validity were verified. Participants’ height was measured without shoes in meters to the nearest millimeter (Wuxi Weigher Factory Co., Ltd., Model TZG, precision 1 mm). Weight was measured by an electronic scale (TANITA Corporation, HD-390, precision 100 g) when participants were barefoot and bareheaded with only light clothes. Blood pressure, fasting, and postprandial blood samples were also tested. Details of this monitoring have been described elsewhere [22].

BMI (kg/m^2^) was calculated as weight in kilograms divided by the height in meters squared. Covariates were as follows. (1) Demographic characteristics including three areas in Shaanxi province, age of participants and education years (total years of schooling); (2) Dietary habits including consumption of red meat, fresh vegetables and fruits, oil, and salt. To assess the intake of red meat, fresh vegetables, and fruits, participants were first asked whether they had eaten the food or not in the last 12 months. If the answer was yes, then the frequency (year, month, week, or day) and amount (g) of each serving was collected. The daily consumption of the above foods was estimated by multiplying the frequency and amount of each time. The daily intake of oil and salt was estimated by the amount of each item a household consumed per day divided by the number of people in the family. (3) Lifestyle factors included cigarette smoking and alcohol intake. Cigarette smoking was defined as a participant who smoked every day during the monitoring period, and alcohol intake as consuming alcohol from any alcoholic beverages (beers or wines) every day, which was collected in the same way as dietary habits. (4) Physical activity including activity at work, during commuting, and in leisure time was divided into three categories (low, moderate, high) according to the Global Physical Activity Questionnaire Analysis Guide.

### Statistical analysis

Initially, BMI and baseline data were reported as mean ± standard deviation and median (25th ~ 75th percentile) for normally and non-normally distributed continuous variables, respectively. They were also tested by t-tests (normally distributed data between two groups), analysis of variance (normally distributed data between three or more groups), or Kruskal–Wallis tests (not-normally distributed data among groups). Categorical variables were described by counts and proportions and examined by χ^2^ tests among groups. The skewness, kurtosis, and normality of BMI were also tested. To determine risk factors potentially associated with BMI distribution, we used QR. Linear regression based on the ordinary least squares (OLS) was used to explain the effects of risk factors on the mean value of BMI. QR could provide a detailed description of the effects on the BMI at any point along its distribution. The choice of BMI percentiles mainly depends on the research objectives and the distribution of the outcome. Nine BMI quantiles of were selected (from 10th to the 90th), step by 10th based on the lowest to the highest BMI value. Coefficients of regression for each quantile were computed and their significance was tested. Multivariate QR models, including demographic characteristics (area, age, sex, and education), dietary habits (red meat, fresh vegetables, and fruits), lifestyle factors (cigarette smoking and alcohol intake), and physical activities, were conducted for each BMI quantile. Coefficients of OLS were also estimated as comparisons. All statistical analyses were performed using R 3.3.1. *P*-values < 0.05 were considered statistically significant in this study.

## Results

### Baseline data for participants

A total of 10,023 out of 10,320 (97.12%) participants were included in the statistical analyses. Among these, 52.25% of males and 53.49% of females were from urban areas, and 57.31% of males and 60.26% of females were from Central Shaanxi. More females (47.11%) had attained ≤6 years of education compared with males (33.57%) (χ^2^ = 192.26, *P* < 0.001). Male participants consumed more red meat (χ^2^ = 14.184, *P* < 0.001) and alcohol (χ^2^ = 34.101, *P* < 0.001) than females, but they showed no difference in consumption of vegetables, fruits, salt, and oil (Table 1).

**Table 1:** Participants’ sociodemographic characteristics (n (%) or mean ± SD)

	Male *N* = 4612(45.37)	Female *N* = 5554 (54.63)	t/χ2	*P*
Residence (urban)	2410(52.25)	2971(53.49)	1.55	0.213
Areas				
North	927(20.10)	867(15.61)	35.04	< 0.001
Central	2643(57.31)	3347(60.26)		
South	1042(22.59)	1340(24.13)		
Age	49.70 ± 14.63	49.40 ± 13.97	1.06	0.29
Education				
≤ 6 years	1546(33.57)	2612(47.11)	192.26	< 0.001
>6 years & ≤ 9 years	2704(58.72)	2621(47.28)		
>9 years	355(7.71)	311(5.61)		
Fresh vegetable/fruit	533.98 ± 444.70	524.65 ± 408.08	1.10	0.27
Red meat	49.20 ± 96.55	26.70 ± 52.24	14.18	< 0.001
Salt	9.53 ± 8.15	9.69 ± 7.96	−0.97	0.33
Oil	56.77 ± 31.71	57.87 ± 31.39	−1.55	0.12
alcohol	35.25 ± 60.35	3.88 ± 17.63	34.10	< 0.001
Smoking everyday	2323(50.37)	55(0.99)	3428.31	< 0.001
Physical activity				
Light	824(17.87)	897(16.15)	12.24	0.002
Moderate	1095(23.74)	1470(26.47)		
High	2693(58.39)	3187(57.38)		

*SD* Standard deviation

### BMI in Shaanxi Province

**Fig. 1:**
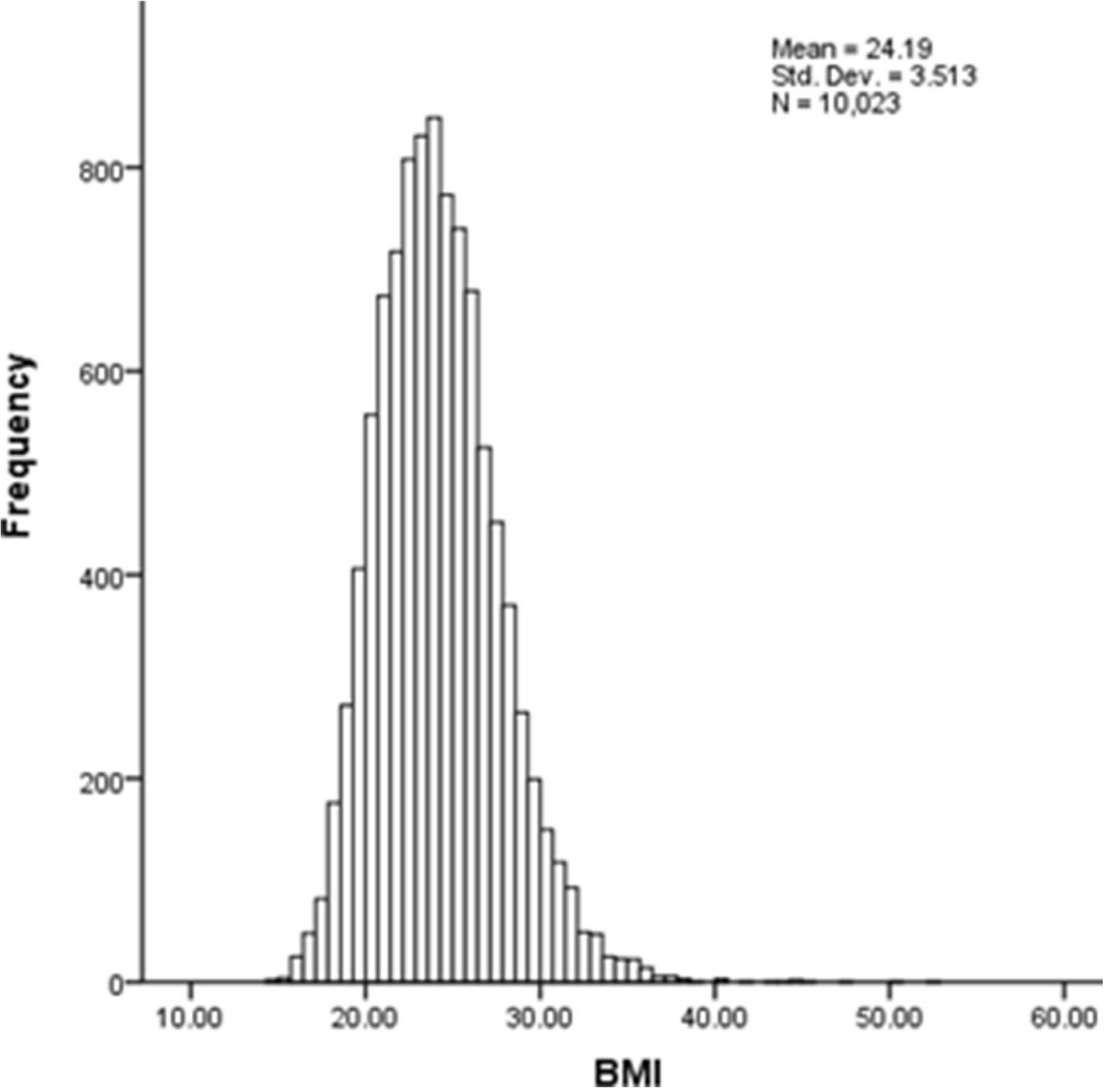
Histogram of BMI in Shaanxi Province. Mean = 24.19 Std.Dev = 3.513 *N* = 10,023

The mean BMI of participants was 24.19 ± 3.51 kg/m^2^, and the range was 14.33 kg/m^2^ to 52.82 kg/m^2^. The 25th, 50th, and 75th percentiles of BMI were 21.68 kg/m^2^, 23.92 kg/m^2^, and 26.31 kg/m^2^, respectively. Statistics for skewness and kurtosis were 0.70 and 1.75, respectively. The normality test showed BMI did not have a normal distribution (Fig. 1). The average BMI of female participants, participants living in urban areas, and participants living in Central Shaanxi were higher than their male, rural, and non-Central Shaanxi counterparts (*P* < 0.001) (Table 2).

**Table 2:** BMI distribution in different socio-demographic characteristics

	Mean ± SD	median(25 ~ 75percentile)	floor	celling	χ^2^/F	*P*
Residence						
Rural	24.00 ± 3.55	23.71(21.49 ~ 26.14)	14.33	50.07	29.466	< 0.001
Urban	24.35 ± 3.48	24.08(21.91 ~ 26.43)	15.63	52.82		
Areas						
North	23.88 ± 3.53	23.51(21.29 ~ 26.05)	14.33	52.82	43.596	< 0.001
Central	24.37 ± 3.58	24.11(21.88 ~ 26.49)	15.31	50.07		
South	23.96 ± 3.28	23.73(21.6 ~ 25.96)	15.64	40.03		
Gender						
Male	24.00 ± 3.36	23.8(21.62 ~ 26.08)	14.33	52.82	15.795	< 0.001
Female	24.34 ± 3.63	24.03(21.73 ~ 26.45)	14.78	50.07		
Education						
≤ 6 years	24.15 ± 3.53	23.87(21.66 ~ 26.19)	14.33	45.05	1.307	0.520
>6 years & ≤ 9 years	24.22 ± 3.5	23.95(21.7 ~ 26.4)	15.63	50.07		
>9 years	24.15 ± 3.47	23.98(21.82 ~ 26.2)	16.13	52.82	3.327	0.190
Physical activity						
Light	24.08 ± 3.67	23.83(21.49 ~ 26.14)	15.31	44.23		
Moderate	24.18 ± 3.64	23.91(21.52 ~ 26.37)	15.63	52.82		
High	24.22 ± 3.41	23.95(21.81 ~ 26.3)	14.33	50.07		

### Risk factors for BMI

**Fig. 2:**
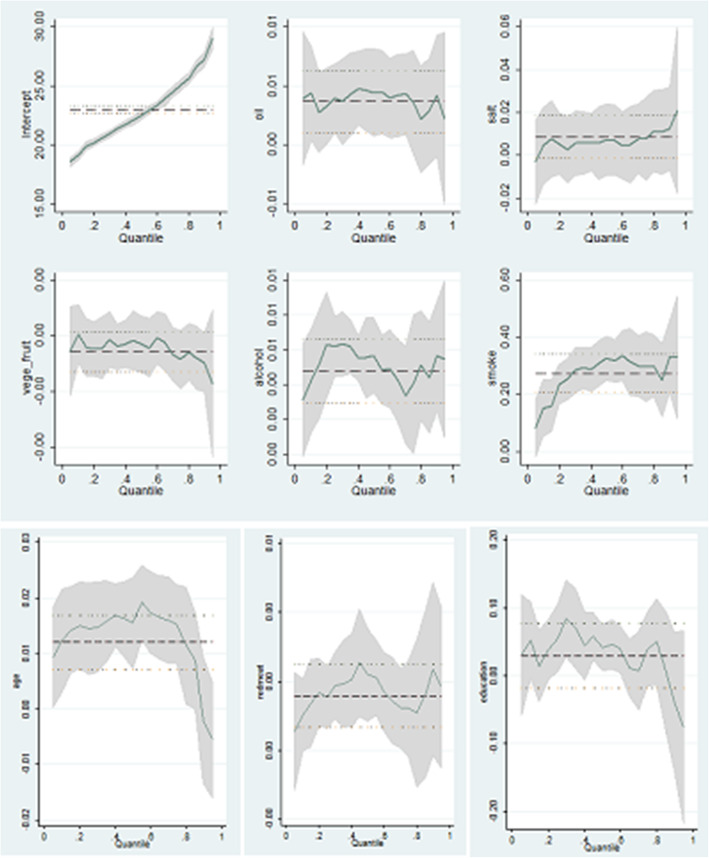
Quantile regression estimates of different variables* The shaded areas around the regression estimates are the 95% confidence intervals. The thick dashed line is the ordinary least squares (OLS) estimate, and the dotted lines show the OLS 95% confidence interval

To determine the potential risk factors for the BMI distribution, multivariate QR analyses of BMI were conducted. Of the covariates associated with all BMI quantiles, age showed a slightly diminished positive association from head to tail of the BMI quantiles. Consumption of oil and alcohol were also positively associated with all BMI quantiles. Cigarette smoking each day was negatively associated with all BMI quantiles and the relationship was *U-*shaped. Compared with light physical activity, high physical activity was least associated with BMI in the 60th quantile. Some risk factors were associated with parts of BMI quantiles. Compared with participants in rural areas, those in urban areas were associated with BMI in low and central quantiles (10th–60th). Females were 0.273–0.594 BMI units higher than males from the center (50th quantile) to the tail (90th quantile) of the BMI quantiles. Participants with 6–9 years of education were 0.23–0.38 BMI units higher for first half of the BMI quantiles compared with those with ≤6 years of education. The positive correlation between red meat and BMI diminished from the 10th to the 50th quantile. OLS analysis found similar results for average BMI (Table 3, Fig. 2).

**Table 3:** estimation parameters of the different factors using quantile regression

covariates^a^	10th	20th	30th	40th	50th	60th	70th	80th	90th	OLS
Residence (urban)	0.356**	0.162	0.248*	0.263*	0.252*	0.271*	0.220	0.240	0.060	0.185*
Areas										
Central	0.071	0.224	0.359*	0.292*	0.485**	0.322*	0.344*	0.263	0.381	0.286*
South	0.261	0.277	0.234	0.024	0.086	−0.106	−0.076	−0.253	−0.284	−0.026
Gender (female)	0.067	−0.038	0.066	0.115	0.273*	0.192	0.343*	0.381*	0.594**	0.261**
Age	0.022**	0.025**	0.024**	0.023**	0.020**	0.021**	0.020**	0.019**	0.001	0.020**
Education										
> 6 years & ≤9 years	0.380**	0.298**	0.314**	0.230*	0.142	0.137	0.215	0.124	−0.250	0.176
>9 years	0.176	0.199	0.137	0.109	0.015	0.100	0.100	−0.028	− 0.341	0.034
Oil	0.005*	0.003	0.006**	0.007**	0.007**	0.007**	0.006**	0.007**	0.006	0.0053**
Salt	0.005	0.005	0.002	0.003	0.005	0.005	0.010	0.007	0.013	0.0075
Red meat	0.002**	0.002**	0.002*	0.002*	0.002**	0.001	0.001	0.001	0.0002	0.0016**
Fresh vegetable/fruit	0.00000	−0.0001	−0.0002	−0.00010	−0.0002	−0.0001	−0.0002	− 0.0003	−0.0002	− 0.0002*
Alcohol	0.003*	0.006**	0.006**	0.006**	0.006**	0.006**	0.006**	0.006**	0.007**	0.0056**
Smoking	−0.617**	−0.837**	−0.896**	− 0.951**	−0.832**	− 0.918**	−0.806**	− 0.642**	−0.545*	− 0.744**
Physical activity										
Moderate	0.010	0.102	0.103	0.100	0.104	0.196	0.311	0.294	0.042	0.154
High	0.692**	0.585**	0.390**	0.336**	0.304*	0.263	0.463**	0.391*	−0.019	0.377**

**β < 0.01,*β < 0.05. a: Reference categories for dummies are as follows: urban: rural; female: male; Central and South: north; > 6 years& ≤ 9 years and >9 years: ≤6 years; smoking daily: not smoking daily; Moderate and high: light

## Discussion

In this study, our QR analysis showed that residence, area, sex, education, and red meat were associated with some quantiles of BMI score. Age, oil, alcohol, smoking, and physical activity were associated with all BMI quantiles. Urban residents tended to have larger scores than rural residents in the central BMI quantiles. Consumption of oil and alcohol were positively associated with BMI throughout the quantiles. Daily cigarette smoking was only negatively associated with BMI. These findings indicated the potential factors that affected the BMI of residents in Northwest China, and suggested in which quantiles of BMI these factors could have an effect.

Sociodemographic factors were found to affect the conditional BMI distribution in previous studies. Evidence from less-developed countries suggested that rural residents weighed less than their urban counterparts [2, 23]. In China, the BMI of residents in urban areas was also higher than that of those in rural areas [24, 25]. These findings agreed with the findings of the present study, but were inconsistent with research from developed countries, in which an inverse association between socioeconomic status and weight was reported [26–28]. A study involving migrants in India reported that there was a rapid increase of adiposity after migrating from rural to urban areas [29]. The higher BMI in urban areas in less developed countries and in rural areas in developed countries may be the result of higher individual- and community-level socioeconomic status. It has been suggested that other factors may cause increased body weight in Northwest China. In this study, sex only played a role at the upper tail of BMI (50th–90th quantile) with females’ BMI higher than that of males, which was consistent with research by Mascietaylor and Beydoun [30, 31]. The BMI of older participants was higher in most BMI quantiles (10th–80th), which suggests public health intervention for overweight and obesity could target the senior population.

The association between diet and BMI has been well studied [32–34]. In this study, we found oil was positively associated with BMI across quantiles. Fresh vegetables, fruits, and salt showed no association with BMI. The statistical insignificance of fresh vegetables and fruits consumption may be unexpected since the higher consumption of fiber-rich food was supposed to be related to lower BMI. However, our QR model included red meat intake, oil consumption, and salt intake. These variables were already controlled for in the main pathway through which vegetables and fruits may influence BMI, contributing to the lack of statistical significance of fiber-rich food.

Alcohol intake was positively associated with the increase of BMI, while daily cigarette smoking had a negative association with BMI quantiles. Similar findings have been shown in studies that reported increasing rates of obesity will be observed if subjects quit smoking [35]. Our QR results showed that the effect of smoking status largely influenced BMI under the 80th percentile, whereas the upper conditional tail of the BMI distribution remained unaffected [36]. The genes relationship or molecular mechanism remains unknown, but a previous study in China showed that smoking was significantly associated with the belief that smoking played an important role in weight control, which could contribute to the understanding of the effect of smoking on BMI quantiles in the Chinese population [37].

There were some limitations in our study. First, we must be cautious in extrapolating the study results to the whole Chinese population as the data are representative of Shaanxi Province. Second, independent variables in this analysis should only be viewed as associated with BMI because this study used a cross-sectional design and cannot make casual inferences. A longitudinal study may be necessary to infer causality. Third, recall bias might have influenced the results, as all information except weight and height were self-reported. Finally, we used data from 2013, and obesity and its risk factors in China are still changing; therefore, an updated study should continue to focus on these associations among Chinese adults.

## Conclusion

Given the shift of BMI distribution, it is not appropriate to examine the whole BMI distribution with means or medians. Instead, methods that could capture the conditional BMI distribution are necessary. This study found that urban residents tended to have a higher BMI score. With the increase of participants’ age, BMI also increased. Education of around 6–9 years and red meat consumption played a positive role only in the first half of the BMI quantiles. With the increased intake of oil and alcohol, BMI increased throughout the quantiles. Smoking daily was the only factor with an inverse association.

## Data Availability

The datasets used and analyzed during the present study are available from the corresponding author on reasonable request.

## Abbreviations

QRs: Quantile regressions
OLS: Ordinary least squares
BMI: Body mass index
